# Nerve growth factor: role in growth, differentiation and controlling cancer cell development

**DOI:** 10.1186/s13046-016-0395-y

**Published:** 2016-07-21

**Authors:** Luigi Aloe, Maria Luisa Rocco, Bijorn Omar Balzamino, Alessandra Micera

**Affiliations:** Institute of Cell Biology and Neurobiology, CNR, Via Del Fosso di Fiorano, 64 I-00143 Rome, Italy; IRCCS - G.B. Bietti Foundation, Via Santo Stefano Rotondo, 6 I-00184 Rome, Italy

**Keywords:** Naïve cell, Tumor cells NGF, NGF-receptors, Cell proliferation, Cell differentiation

## Abstract

Recent progress in the Nerve Growth Factor (NGF) research has shown that this factor acts not only outside its classical domain of the peripheral and central nervous system, but also on non-neuronal and cancer cells. This latter observation has led to divergent hypothesis about the role of NGF, its specific distribution pattern within the tissues and its implication in induction as well as progression of carcinogenesis. Moreover, other recent studies have shown that NGF has direct clinical relevance in certain human brain neuron degeneration and a number of human ocular disorders. These studies, by suggesting that NGF is involved in a plethora of physiological function in health and disease, warrant further investigation regarding the true role of NGF in carcinogenesis. Based on our long-lasting experience in the physiopathology of NGF, we aimed to review previous and recent in vivo and in vitro NGF studies on tumor cell induction, progression and arrest. Overall, these studies indicate that the only presence of NGF is unable to generate cell carcinogenesis, both in normal neuronal and non-neuronal cells/tissues. However, it cannot be excluded the possibility that the co-expression of NGF and pro-carcinogenic molecules might open to different consequence. Whether NGF plays a direct or an indirect role in cell proliferation during carcinogenesis remains to demonstrate.

## Background

The Nerve Growth Factor (NGF) was discovered by R. Levi-Montalcini nearly 60 years ago after the transplantation of a malignant mouse sarcoma into the body wall of a 3-day-old chick embryo [1, 2]. Subsequent studies revealed that the purified murine NGF (adult submaxillary gland) stimulates morphological differentiation, regulates neuronal gene expression (through interaction with specific cellular receptors) and plays a critical role in mature neurons for acting directly on peripheral sensory and sympathetic neurons and for maintaining their function and phenotype [3, 4]. Structural, biochemical and molecular studies indicate that a trophic interaction failure between target cells and their innervations might result in nerve dysfunction and neuronal degeneration [5, 6]. These findings led to the hypothesis that purified NGF might be a useful tool to prevent and/or protect peripheral nerves from degeneration, as observed in Diabetes [7]. The history of NGF in clinical trials of Diabetes is exemplary with respect to the potentiality of NGF in the care of peripheral neuropathies [8, 9]. Moreover, studies carried out in animal models and humans demonstrated that NGF can promote survival, differentiation and functional activity of peripheral sensory and sympathetic nerve cells [8]. Diabetes is a metabolism disorder characterized by degeneration of peripheral neuron/fibers and altered local levels of NGF/NGF receptors and deregulation of NGF signal pathway [7]. In experimental models of diabetic neuropathies, NGF administration reversed the neurodegenerative signs and normalized the activity of neurons belonging to the Peripheral Nervous System [6]. The results of the above reported clinical trials were partially confirmed by succeeding clinical trials and thereafter the human studies were closed [8]. The reason of dissimilar outcomes between first and second clinical trials is still not clear. A possible hypothesis might encompass a different biological preparation and/or composition of NGF formulation, the not-homogeneous study populations (in terms of age, onset and severity as well as clinical history of the neuropathy), the different selection of the placebo patient group and finally the occurrence of undesirable side effects [10]. The most reasonable explanation for this clinical study failure and the interruption of NGF investigations in diabetic neuropathies could be associated with the necessity to use low NGF dosage (for side effects) in comparison with those of animal studies [10]. The Authors concluded that a simply approach to investigate the role of NGF in human peripheral neuropathy could be the use of molecules with the ability to stimulate both synthesis and release of NGF at the proximity of damaged tissue [10]. This aspect would imply the possibility to induce endogenous NGF upregulation, with no NGF-related side effects [10]. Subsequently, studies revaled that NGF exerts a critical protective action on specific brain cells and particularly on the basal forebrain derived neurons undergoing degeneration in Alzheimer disease (AD) [5] and a variety of non-neuronal and neoplastic cells [1]. Moreover, these studies revealed that the protective NGF role in human target cells might occur also outside the classical nervous system domain, as observed in the treatment of corneal ulcers [11], Glaucoma [12], Maculopathy [13], Retinitis Pigmentosa [14] and AD [15, 16]. These studies would suggest the use of NGF in the near future for the treatment of human pathologies with damaging of NGF-responsive cells.

By the way, the presence of NGF and NGF-receptors in cancer cells raised the question as whether NGF is involved in promoting cell proliferation and eventually cancer cell survival [17]. To gain further information on this aspect, our aim was to summarize and review our and other literature available finding on NGF in cancer cell survival, proliferation and cell arrest, within and outside nervous system, both at baseline and following exposure to purified NGF.

### NGF as pro-survival molecule

**Fig. 1:**
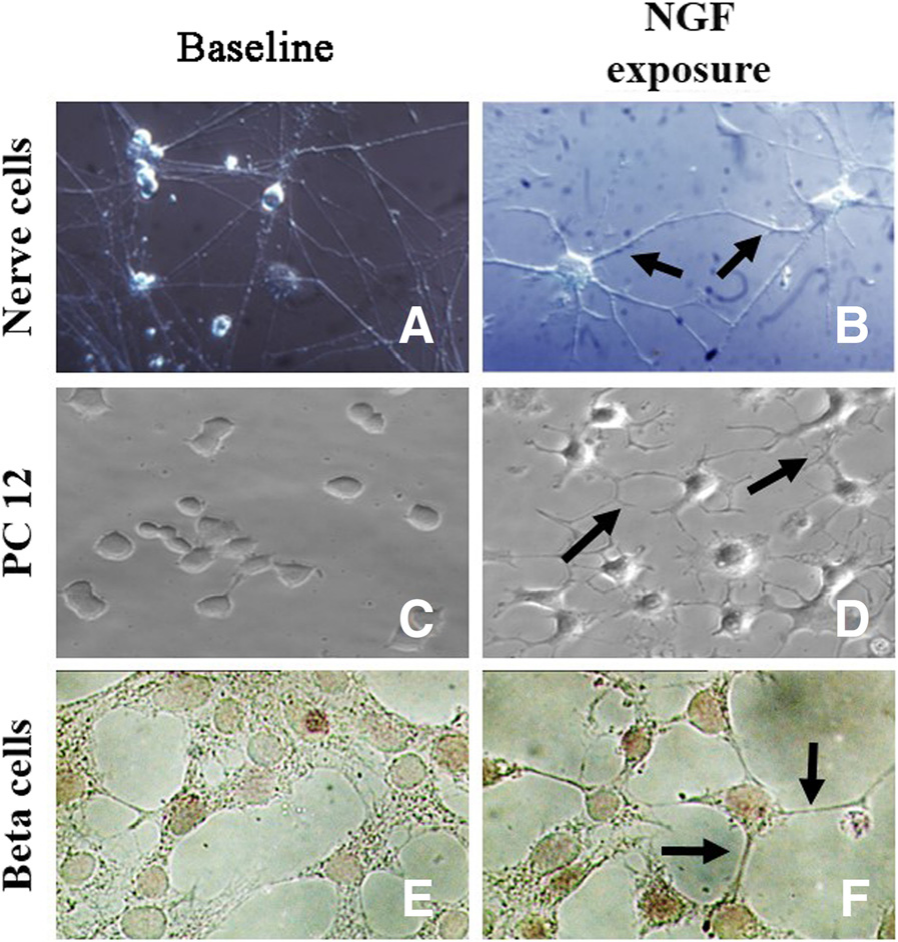
Photographic illustration of untreated (baseline, *left panels*) and treated (NGF exposure, *right panels*) cultured cells. As shown by phase contrast acquisition, exposure to 20 ng/ml NGF for seven consecutive days promoted differentiation and neuritis outgrowth (*right panels*) rather than cell proliferation of sympathetic (**b**), tumor PC-12 (**d**) and beta pancreatic (**f**) cell lines, as compared to untreated ones (*left panels*). Magnifications: x400. **a**-**d**, phase contrast; **e**-**f**, light microscopy

NGF and NGF-receptors (trkA^NGFR^ and p75^NTR^) play a critical role in proliferation, differentiation and survival of developing peripheral and central nervous system neurons, influencing their activity in many ways [2, 18–20]. Focused in vitro studies showed that rat sympathetic nerve (Fig. 1a) and brain cells (subvetricular zone) exhibited both differentiation and neuritis outgrowth but no cell proliferation when cultured in the presence of purified NGF (Fig. 1b, arrows). The absence of cell proliferation upon NGF exposure is consistent with several studies showing that systemic NGF administration is associated with an increased activity of NGF at both peripheral and central target neurons [2, 4, 6, 19]. An interesting protective role of NGF is the ability to guarantee the physiological activity inside the tissue microenvironment, by preserving the tissue/organ functional activity, as observed in the protection of corneal nerve cells and in the regulation of homeostasis within nervous, immune and endocrine systems [21]. In vivo and in vitro studies confirmed that NGF plays a marked role in the (i) differentiation and (ii) survival of developing neurons belonging to peripheral and central nervous system as well as in the (iii) protection of degenerating young and adult neurons [2]. Also, NGF has been reported to promote the regulation of neurotransmitter expression/release, facilitate axon guidance/synapse formation and modulate synaptic activity/function (for further details see [4]). Overall, these findings are consistent with other studies showing that systemic NGF administration is associated with an increased biological activity of NGF-target cells and not related with the induction of cancer cell proliferation [4, 17, 22, 23].

### NGF and uncontrolled cell proliferation

The question about NGF role in tumor induction/progression has been investigated in different in vivo and in vitro experimental approaches. Evidences that some tumor tissues and their isolated tumor cells can express NGF-receptors and/or store NGF protein suggested the hypothesis that NGF might be involved in tumor cell induction and/or progression [24–27]. A consistent number of experimental studies have demonstrated the expression of NGF and NGF-receptors in neural crest-derived cells as well as in tumor cell lines from neuroblastoma, lymphoma, glioma, medulloblastoma adrenal tumors and melanoma, suggesting that NGF administration on trkA^NGFR^-bearing cells could lead to cell differentiation and improved prognosis [28–30]. On the contrary, NGF by itself is unable to induce/generate cancer cell proliferation from normal tissues/cells or sustain cancer cell progression [31]. Therefore, the hypothesis that NGF administration can promote uncontrolled cell proliferation (leading to cancer development) seems to have weak experimental evidence. It is noterworth to highlight that consistent NGF amounts are physiologically produced, stored and secreted by several cells/tissues as well as released into mammalian bloodstream (including humans) [2, 32, 33]. Several tissues produce and release NGF under physiological and neoplastic conditions (prostate included) and the locally-released NGF can exert both differentiatial and pro-survival activities on neuronal and non-neuronal tumor cells, depending on type of tumor and expression of trkA^NGFR^ and/or p75^NTR^ receptors [34, 35]. Cell proliferation, survival and differentiation are under control of different signal transduction systems. The Mitogen-activated protein kinases (MAPKs) and the Ras (Ras/Raf/MEK/ERK) cascades represent key molecules for trk signaling and modulate all the hallmarks of cancer cells (survival, migration and invasion). Thereafter, specific inhibitors targeting these pathways represent crucial approaches to counteract tumor developing/progression, as observed for the inhibitor of MEK1 (Cobimetinib) allowing the differentiation and apoptosis in neuroblastoma cells and the kinase inhibitor D11 mediating apoptosis of cancer cells resistant to chemotherapy [36, 37]. On the other side, growth factors modulate several aspects of cell functions inside different microenvironments. The presence of a stem-like phenotype in tumors was confirmed by the identification of a small portion of cell population with the characteristics of stem cells inside tumor tissues [38]. Because of the self-renewing capacity and the multi-directional differentiative potential, these cancer stem cells can represent source of tumor cells with different degree of differentiation inside the tissue. Contrasting data encompass the relationship between CD133/nestin (markers of neural stem cells and of cancer stem cells in neurogenic tumors) and prognosis of patients with glioma [38]. Therefore, it is undoubted the great value of understanding the role of growth factors, and merely NGF and NGF-receptors, in tumor developing/progression, as well as the development of tumor growth factor targeted approaches.

Merely to NGF interaction in tumor cells, Zhu and coworkers highlighted that the NGF–trkA^NGFR^ interaction influences growth and spread of pancreatic cancer cells, Zhang and coworkers highlighted the prognostic value of NGF-receptors while Missale and coworkers showed the NGF-receptor overexpression associated with a good prognosis [39–41]. Farina and coworkers described a different trkA^NGFR^ isoform (trkA III, the result of an alternative splicing) able to induce an aberrant and aggressive proliferation in neuroblastoma cells [42]. In a very recent study, Ruggeri and co-workers highlighted that NGF binding to trkA^NGFR^ and TRAIL (TNF-related apoptotis-inducing ligand) might suppress neuroproliferation in neuroblastoma by inducing apoptosis [43]. These findings suggest that the high trkA^NGFR^ expression can provide a more favorable survival prognosis in breast cancer and neuroblastoma, although the underlined mechanisms and the direct relationships between trkA^NGFR^ and p75^NTR^ remain poorly understood and/or explored. In addition, it was reported that the selective p75^NTR^ expression by prostate tumor cells could induce cell cycle arrest and apoptosis, both crucial steps for potential anti-tumoral therapy [34, 35]. Studies reported by Ødegaard and coworkers showed a reduced expression/activation of trkA^NGFR^ in effusions as compared with solid ovarian carcinoma and that trkA^NGFR^ expression appeared to be independent of cell cycle progression, suggesting the phosphorylated trkA^NGFR^ form (known as p-trkA^NGFR^) as a potential marker of prognostic value [30, 39].

### NGF and tumor cell inhibition

Over the last three decades, a consistent number of published studies have shown that NGF can promote cell differentiation and arrest tumor progression, as observed in primary and cell line tumor cells [31, 44–50]. Particularly, several in vitro experiments have shown that NGF is capable of retarding growth and inducing persistent differentiation of neurogenic tumor cell lines [51]. As shown, PC-12 tumor cells proliferate under baseline conditions (Fig. 1c) and start to differentiate and produce neuritis after NGF exposure over few consecutive days (see arrows in Fig. 1d). Likewise, cultured beta-tumor pancreatic cells proliferate in the absence (Fig. 1e) and stop to proliferate after the addition of NGF to the culture medium (arrows in Fig. 1f). Studies on animal models demonstrated that NGF is able to (i) induce the persistent reduction of the number of Ethylnitrosourea (ENU) -induced neurinomas and (ii) increase the survival time of rats after intracerebral implantation of the F98 anaplastic glioma cells [52]. Either in tranplacental or postnatal ENU-exposure, NGF treatment caused a reduction of the number of ENU-induced neuromas in rats [52, 53]. As observed after topical administration, exogenous NGF can arrest tumor cell proliferation in human ocular glioma, prompting to the hypothesis of an inhibitory rather than promoting NGF effect on cancer cell (stimulation and tumor progression) [46, 54, 55]. These observations seem to support the hypothesis that the predominant expression of trkA^NGFR^ might facilitate cell differentiation while the p75^NTR^ expression in the absence of trkA^NGFR^ might facilitate cell proliferation [56]. Regarding the hypothesis of a favorable link between expression of NGF/NGF-receptors and tumor cell proliferation, it should be taken into consideration that the presence and/or release of “well established” pro-oncogenic molecules might precede the presence of NGF. These studies indicated that (i.) the NGF treatment can trigger the development of a more differentiated cell phenotype and as result cause the reduction or complete cessation of tumor growth and more interesting (ii.) the effects of NGF can be persistent, all together supporting the hypothesis that NGF can reverse transformed properties of susceptible tumor cell progression.

### NGF, microenvironment and immunocompetent cell

The interaction between immune and tumor cells is crucial for tumor growth and progression, as highlighted by several old and recent studies in both animal and human models [47–49]. Although tumors have their own “cell shelter mechanisms” (immune escape, resistance to apoptosis and cell survival), a host-mediated immune response against tumors can occur and consequently two different models have been proposed. The “immunosurveillance model” suggests that tumor cells (by expressing proper surface antigens) are regarded as “non-self” and thereafter eliminated by the immune system, although some unknown mechanisms counteract with this physiological protective route [48]. According to the “danger model”, the professional sentinels of tissue damage (Antigen Presenting Cells, APCs; dendritic cells and activated macrophages), B and T cells are activated/stimulated by risk-signals but do not recognize cancer cells as dangerous, and thereafter the appropriate T cell response to tumors does not occur [48].

In both cases, the composition of tissue microenvironment might play a crucial role. An accurate analysis of the microenvironment in a variety of solid tumor sections has revealed the presence of a T cell–infiltrated phenotype, macrophages, neutrophils, recruited mast cells and infiltrating eosinophils [49]. Such a microenvironment might play a crucial role in tumor launch/progression, including the local sustaining and development of tumor-associated angiogenesis [49]. Microenvironment is strictly linked to the genetic background and the interplay between infiltrating/resident immune (APCs, NK cells, B-T lymphocytes as well as mast cells and eosinophils) and epithelial/stromal cells. The release of different pro/anti-inflammatory and pro/anti-angiogenic factors (cytokines, chemokines, growth/fibrotic/angiogenic factors and tissue remodeling enzymes) might significantly influence and/or modulate local immune response and angiogenesis [48, 50, 57–59].

Studies published during the early ’90s revealed that NGF plays a critical role in the mechanisms of neuro-immune-endocrine homeostasis [60]. The first study prospecting this aspect reported that NGF stimulates the survival of Mast Cells (MCs) and modulates the specific function of MCs and lymphocytes, providing substantial evidences that NGF could actively contribute to both innate and adaptive immune responses [61, 62]. Further studies into this field led to the discovery that macrophages, granulocytes, T and B subtypes, NK cells and eosinophils are not only NGF-target cells (survival and function) but also synthesize, store and release consistent amounts of NGF [63–68]. The presence of physiological amounts sustains the crucial NGF role in both innate and adaptive immune cells. The tumor microenvironment might release high amount of NGF and respond to extracellular NGF in autocrine/paracrine fashion. NGF immunoreactivity has been observed in several tumor tissues and cells [69, 70]. The observation that NGF is a soluble mediator, either released into or produced by the tumor microenvironment, and the fact that NGF is able to act on certain immune-cell activities, would suggest a possible control of cell proliferation towards resistance to cancer survival [71, 72]. It is noteworthy to highlight that some soluble mediators can exert a dual-faced action, implying that immune cells can have both active and/or bystander effects [71, 72].

### Conclusions

**Fig. 2:**
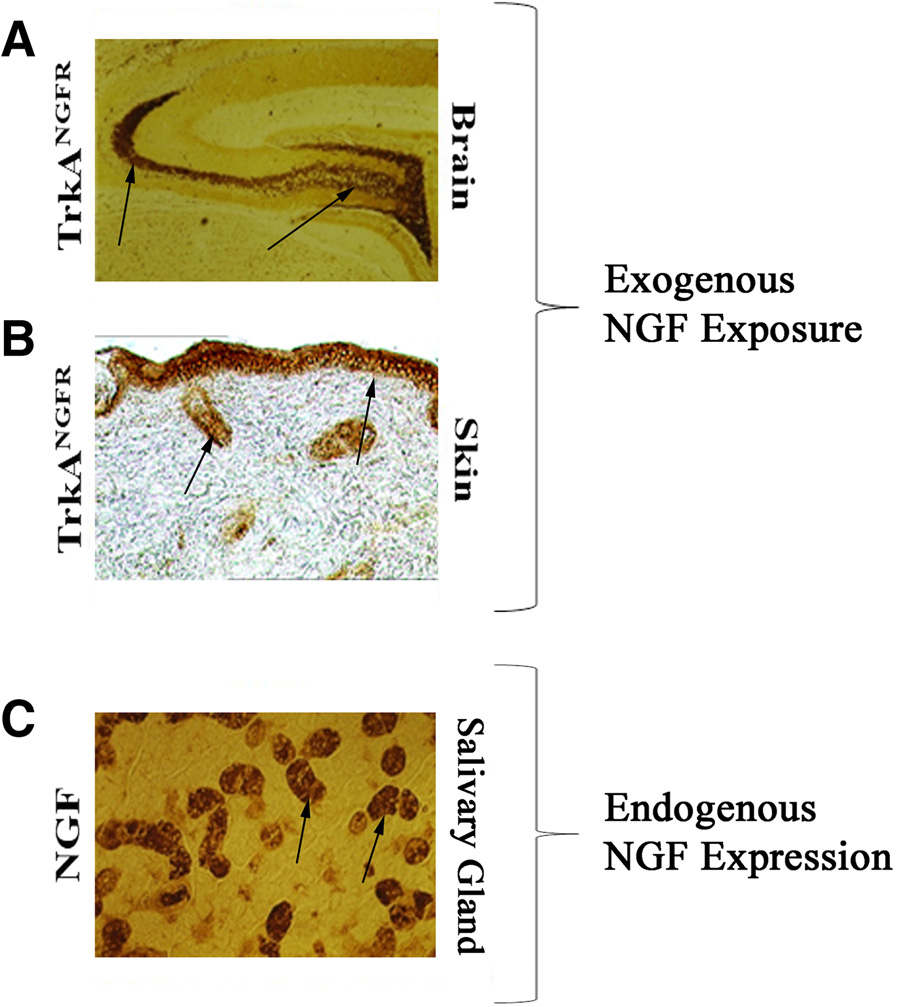
High levels of exogenous and/or endogenous NGF in tissues without cell proliferation. Illustrations of exogenous NGF-induced trkA^NGFR^ expression (**a**-**b**) and endogenous NGF expression (**c**). **a** Representative brain section from a young rat treated with 1 μg purified NGF into the third brain ventricle. Note the trkA^NGFR^ immunreactivity in the dentate gyrus of the hippocampus (*arrows*). The absence of any sign of cell proliferation within and nearby the hippocampal tissues is clearly visible. **b** Representative cutaneous tissue section from a mouse exposed to subcutaneous administration of 10 μg purified NGF. An increased trkA^NGFR^ immunoreactivity is visible (*arrows*) in the dermal tissue having no cell proliferation. **c** Representative submaxillary gland sections from a 10-week-old male-mouse probed with anti-NGF antibody (*arrows*). Despite the massive NGF immunoreactivity in murine salivary gland (tubular cells; see *arrows*), no cell proliferation nor cell neoplasy characterized the gland tissue. Magnifications: **ab**, x100; **c**, x400, light microscopy

NGF is released in the bloodstream of mammalians (human included) and is critically involved in the protection of several neuronal and non-neuronal cell types, including healthy and tumor cells [2, 73]. The presence of NGF protein and the expression of NGF-receptors in cancer cells have produced a number of divergent hypotheses as whether NGF is directly involved in cancer cell proliferation and differentiation. Since the great heterogeneity of cancer cells (stage of differentiation, malignancy and production/release of different ligands) may represent a signal for promoting cell neoplasy, the identification of NGF – cancer cell interaction might clearly establish whether this factor acts as a first signal. So far, these aspects have not been taken into consideration or not yet been sufficiently investigated. The observation that NGF-exposed naive cells do not generate cancer cell phenotypes argues against a primary role of NGF in promoting cancer cell generation. Indeed, the experimental evidence that NGF exposure induces differentiation rather than proliferation in cancer cell line (pheochromocytoma, glioma, neuroblastoma and pancreatic beta cells) argues against the pro-cancer role of NGF (Fig. 2ab). The observations that (i) the murine salivary glands, by producing and releasing great amounts of NGF (Fig. 2c), do not induce local/systemic tumor cell development; (ii) the exogenous single or repeated (systemically or intracerebroventricular) administration of NGF is unable to generate local tumor development [7, 8, 11–13]; (iii) the large amount of NGF produced by Sarcomas’ 180 and 137 were not able to generate tumor cell proliferation when transplanted into chick and mouse embryos, but on the contrary stimulated nerve cell differentiation and neuritis outgrowth [17, 22] and (iv) the evidence that the addition of purified NGF to tumor cell lines (rat PC-12) [45] or human epindenoma and glioma [31] can arrests tumor cell proliferation and stimulate cell differentiation, support the hypothesis that the NGF administration by itself is not sufficient to generate/promote cell tumorigenesis, including cancer cell proliferation and tumor development/progression. Likewise, recent studies indicate that topical ocular NGF administration reduces glioma in vivo and the progression of pediatric optic glioma [46, 54, 55]. The possibility that NGF in concert with other pro-cancer biological mediators might play a role in cell survival cannot be excluded, but this effect and the underlined mechanisms need to be identified. In this contest, it is worth mentioning that NGF is produced at high concentrations in human prostate, under physiological and neoplasy conditions [35]. Cumulatively, the above reported in vitro and in vivo observations sustain the hypothesis that persistent NGF activity can suppresses cancer cell proliferation, even if these neoplastic cells express both NGF-receptors and respond to NGF action.

This hypothesis is consistent with a number of previous and recent studies showing that NGF promote differentiation of cultured tumor cells and that single/repeated in vivo subcutaneously [17, 22, 72, 73], intracerebrally [5, 74], intranasally [75], topically [11–13, 76–79] and orally [80] NGF administered or even endogenously-induced/released NGF [73] do not cause uncontrolled cell proliferation nor lead to cancer cell generation. Although the available observations are consistent with such hypothesis, further studies might be necessary to determine whether a given population of tumor cells is entirely NGF-responsive or contains a proportion of unresponsive cells.

Finally, in line with the observation that repeated NGF administration in human pathologies do not promote cell neoplasy [17, 46, 54, 55], our working hypothesis and future investigation are to pursue studies on biochemical and molecular signals and factors, as well as to develop novel in vitro and in vivo strategies to confirm or debate the anti-tumoral properties of NGF.

## Abbreviations

AD, Alzheimer disease; APCs, antigen presenting cells; ENU, EthylNitrosoUrea; MCs, mast cells; NGF, nerve growth factor; NK, natural killer cells; p75^NTR^, neurotrophin receptor (low affinity NGF receptor); PC-12, PheoChromocytoma cell line derived from rat adrenal medulla; trkA^NGFR^, Tyrosine Kinases Receptor of NGF
